# Dynamics of virological suppression and systemic immune-inflammatory response following antiretroviral therapy in pediatric HIV infection

**DOI:** 10.3389/fcimb.2026.1918829

**Published:** 2026-09-15

**Authors:** Mavera Uşaklıoğlu Erol, Behiye Benaygül Kaçmaz, Ayper Somer, Selda Hançerli Törün

**Affiliations:** 1 Department of Pediatrics, Division of Pediatric Infectious Diseases, Istanbul University, Istanbul Faculty of Medicine, Istanbul, Türkiye; 2 Department of Pediatrics, Division of Pediatric Infectious Diseases, University of Health Sciences Türkiye, Istanbul Training and Research Hospital, Istanbul, Türkiye

**Keywords:** antiretroviral therapy, CD4, CD4/CD8, HIV, immune activation, inflammation, pediatric, systemic immune-inflammation index (SII)

## Abstract

**Background:**

Persistent systemic inflammation remains a hallmark of treated HIV infection and is not reliably resolved by virological suppression alone. The Systemic Immune-Inflammation Index (SII), a composite hematologic index [(neutrophil × platelet)/lymphocyte count], has emerged as a candidate inflammatory biomarker. Longitudinal SII data following antiretroviral therapy (ART) initiation in pediatric HIV are lacking.

**Methods:**

This single-center retrospective paired longitudinal study included 30 pediatric patients with HIV with laboratory data at baseline and 12 months post-ART. The primary outcome was change in SII; secondary outcomes included CD4%, HIV RNA, and double-negative T-cell percentage (DNT%). Non-improvers were characterized by age, sex, ART regimen, nutritional status, and co-infection status. Paired comparisons used the Wilcoxon signed-rank test; Spearman correlation was the primary inferential method, with Pearson as a sensitivity analysis.

**Results:**

Median SII declined numerically (342,637 to 250,527) but did not reach significance (*p* = 0.428), despite significant improvement in CD4% (*p* < 0.001) and HIV RNA (*p* < 0.001). SII failed to improve in 13/30 patients (43.3%). The baseline inverse correlation between SII and CD4% (*r* = −0.450, *p* = 0.013) was substantially attenuated at month 12 (*r* = −0.004, *p* = 0.985). A parametric correlation between ΔSII and ΔHIV RNA (*r* = +0.534, *p* = 0.002) was not confirmed on rank-based testing (ρ = +0.004), reflecting leverage from two extreme-viremia patients. The 1–2-year age group had the highest rate of SII non-improvement (75.0%); an unadjusted *post-hoc* comparison with the 6–11-year group yielded a nominal *p* value of 0.038. Female sex was more frequent among non-improvers (53.8% vs. 17.6%; *p* = 0.056), although the difference was not statistically significant. Genvoya^®^ use showed a similar non-significant trend (*p* = 0.132). Nutritional status, herpesvirus co-infection, and virological suppression at month 12 were not significantly associated with SII trajectory.

**Conclusion:**

In this pediatric HIV cohort, SII failed to improve in nearly half of patients despite successful ART, dissociating from both CD4 reconstitution and HIV RNA suppression. These findings suggest SII reflects a residual inflammatory state driven by mechanisms beyond viral replication, including ART class-specific, sex-related, and developmental factors. Routine SII monitoring may identify patients at ongoing inflammatory risk despite virological success; larger prospective studies are needed.

## 1 Introduction

The advent of combination antiretroviral therapy (cART) has fundamentally altered the natural history of HIV infection, converting what was once a uniformly fatal disease into a manageable chronic condition with near-normal life expectancy for those with sustained access to treatment (Deeks et al., 2013a). Nevertheless, persistent immune activation and systemic inflammation remain hallmark features of treated HIV infection and are not reliably resolved by plasma viral suppression alone (Deeks et al., 2013b; Lederman et al., 2013). These residual inflammatory abnormalities have been causally implicated in non-AIDS-defining morbidities — including cardiovascular disease (Crowe et al., 2010; Freiberg et al., 2013), neurocognitive dysfunction (Saylor et al., 2016), accelerated ageing (Pathai et al., 2014; Chiappini et al., 2018), and end-organ damage (Deeks et al., 2013b) — and are increasingly recognized as major determinants of long-term outcomes in virologically suppressed individuals. Their pathogenesis is multifactorial, encompassing microbial translocation across a disrupted gut epithelial barrier, persistent antigen stimulation from tissue-associated viral reservoirs, chronic co-infections, and long-standing dysregulation of both innate and adaptive immune compartments (Brenchley et al., 2006; Hunt, 2012).

The challenge of monitoring residual inflammation in clinical practice has renewed interest in composite hematologic indices derivable from standard complete blood counts, which are universally available, inexpensive, and obtained as part of routine HIV care. Among these, the Systemic Immune-Inflammation Index (SII) — defined as (neutrophil count × platelet count)/lymphocyte count — has emerged as a candidate biomarker integrating multiple immune cell lineages into a single numerical score. In oncology, SII has been validated as a prognostic marker across numerous tumor types (Hu et al., 2014; Wang et al., 2020; Li et al., 2023; Panettieri et al., 2024); its application in infectious disease contexts is more recent but growing. In the HIV literature, SII has been associated with mortality in HIV-associated cryptococcal meningitis in adult patients (Zhao et al., 2025) and with postoperative surgical site infection in adult HIV-positive surgical patients (Gong et al., 2026), and with elevated cardiovascular risk, including hypertension, in adult people living with HIV (Ou-Yang et al., 2023).

However, the utility and interpretability of SII in treated HIV infection — and particularly in pediatric HIV — remain incompletely characterized. In a cross-sectional study comparing people with and without HIV, Wang et al. recently found that virologically suppressed people with HIV had lower SII values than HIV-negative individuals, suggesting that SII in this setting reflects HIV- and ART-related hematologic changes more than it reflects true inflammatory burden (Wang et al., 2025). These data underscore that SII in the context of HIV does not map straightforwardly onto conventional immunological or virological endpoints and must be interpreted within its specific biological context.

Critically, no longitudinal data exist on how SII evolves following ART initiation in children or adolescents with HIV, and it is unknown whether SII trajectories in the pediatric setting parallel virological suppression or CD4 reconstitution in a manner analogous to adults. This gap is particularly consequential given that immunological immaturity, developmental variation in hematologic reference ranges, predominantly vertical HIV transmission, and ART-related hematologic effects may collectively shape SII dynamics in children in ways that are not predictable from adult data (Carr, 2000; Dame and Sutor, 2005; Kollmann et al., 2017; Makoni et al., 2016). A small number of studies have suggested that related inflammatory indices may help identify patients at risk for suboptimal immune reconstitution following ART initiation (Drozd et al., 2016; Yu et al., 2025), but longitudinal SII data in children and adolescents with HIV are lacking.

The present study was therefore designed to address this gap. Using paired baseline and 12-month measurements obtained from a pediatric HIV cohort initiating ART, we evaluated longitudinal changes in SII and examined their relationship with concurrent changes in plasma HIV RNA, CD4 percentage, and double-negative T-cell (DNT%) percentage. Particular emphasis was placed on characterizing the subset of patients in whom SII failed to improve despite virological and immunological treatment success, and on identifying clinical, virological, hematological, nutritional, and regimen-specific factors associated with divergent SII trajectories. To our knowledge, this represents one of the first longitudinal analyses of SII dynamics following ART initiation in a pediatric HIV cohort.

## 2 Materials and methods

### 2.1 Study design and patient population

This was a single-center retrospective paired longitudinal study of pediatric patients with confirmed HIV infection who had available laboratory data at two time points: immediately prior to ART initiation (baseline) and at 12 months after ART initiation (month 12). Patients were eligible for inclusion if they had evaluable paired measurements for the Systemic Immune-Inflammation Index (SII) and at least one additional variable of interest (CD4 percentage, DNT percentage, or plasma HIV RNA). Patients with missing data at either time point that precluded calculation of SII were excluded from SII-specific analyses but retained in analyses of other variables where data were available. Clinical records were retrospectively reviewed between September 2025 and March 2026. Of 40 patients with HIV infection identified during this period, 10 were excluded: 6 due to incomplete clinical records precluding extraction of baseline variables, and 4 due to absence of 12-month laboratory data in the institutional database. The remaining 30 patients with complete paired data constituted the primary analytic cohort (**Figure 1**). Consecutive eligible patients were included; no additional selection was applied beyond the availability of complete paired laboratory data. The 12-month assessment time point was defined with a tolerance window of ±2 months (i.e., blood samples obtained between 10 and 14 months after ART initiation were considered evaluable). This study was not powered *a priori*; the sample size of 30 represents the complete population of eligible patients identified at our center during the data collection period. The study was conducted in accordance with the principles of the Declaration of Helsinki and applicable institutional data governance requirements. Ethical approval was obtained from the Institutional Review Board (Number: 2026/831), and informed consent was obtained from parents or legal guardians of all participants.

**Figure 1:**
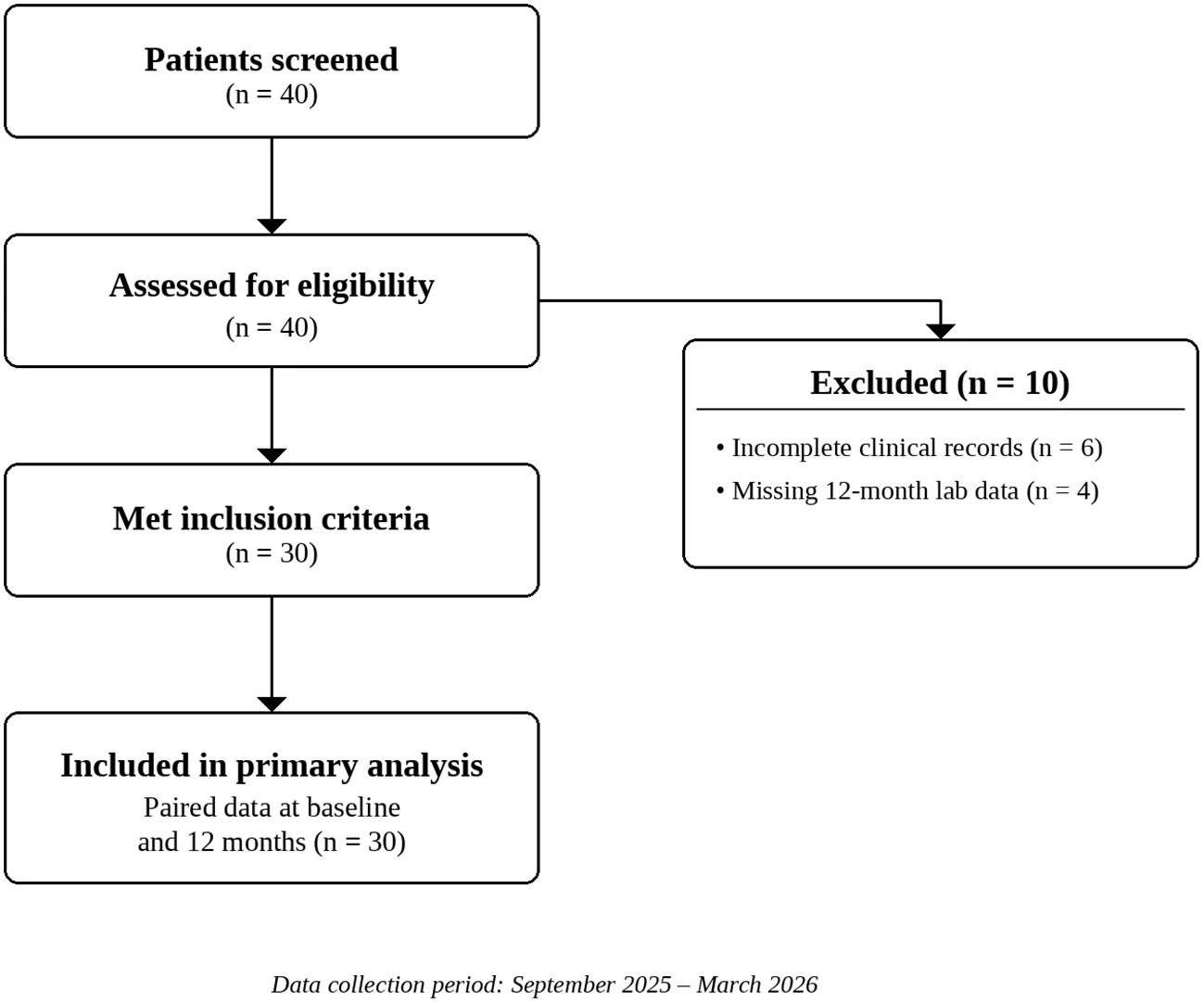
Patient flow diagram. Of 40 patients with HIV infection screened between September 2025 and March 2026, 10 were excluded (6 due to incomplete clinical records, 4 due to missing 12-month laboratory data), leaving 30 patients included in the primary analysis.

### 2.2 Data structure and variable extraction

The primary outcome variable was SII, calculated as (absolute neutrophil count [ANC] × platelet count)/absolute lymphocyte count (ALC), using absolute cell counts per µL as reported by the clinical laboratory. Secondary variables included CD4 percentage (CD4%), double-negative T-cell percentage (DNT%), and plasma HIV RNA (copies/mL). Additional variables examined as potential determinants of SII trajectory included age, biological sex, body mass index (BMI), nutritional status assessed by the Gomez classification, ART regimen, route of HIV transmission, immunological disease stage, co-infection status with cytomegalovirus (CMV) and Epstein-Barr virus (EBV) as determined by quantitative real-time PCR (DNA viral load measurement), and valganciclovir use. In accordance with institutional nutritional support protocols, patients newly diagnosed with HIV who had a Gomez score below the normal threshold (weight-for-age <90%) received supplemental enteral nutritional support as part of their standard clinical care.

### 2.3 Laboratory methods

Peripheral blood lymphocytes were immunophenotyped by flow cytometry using a Navios EX flow cytometer (Beckman Coulter, Brea, CA, USA). Measured parameters included CD3^+^, CD4^+^, and CD8^+^ T-cell counts (cells/µL), the CD4/CD8 ratio, and double-negative T cells (CD3^+^CD4^-^CD8^-^ [DNT], percentage and absolute count). Plasma HIV RNA was quantified by real-time PCR using the COBAS HIV-1 assay on the COBAS 6800 platform (Roche Diagnostics). The lower limit of quantification was 20 copies/mL. HIV RNA values were extracted from the numerical results reported by the clinical laboratory at the time of testing. EBV and CMV DNA viral loads (copies/mL) were measured by real-time PCR using the Roche COBAS assay on the same COBAS 6800 system. Complete blood count parameters — including absolute neutrophil count, platelet count, and absolute lymphocyte count — were obtained from routine automated hematology analyzers and used for SII calculation as described in section 2.2.

### 2.4 Handling of HIV RNA values

Plasma HIV RNA values reported below the lower limit of quantification of the assay (i.e., “<20 copies/mL”) were assigned a numerical value of 10 copies/mL for computational purposes, consistent with standard practice for censored virological data. Historical laboratory result reported as >10,000,000 was assigned 10,000,000. These assignments enabled parametric and rank-based analyses on the full dataset without introducing analytic bias, while appropriately flagging that the Pearson correlation, which is sensitive to extreme values, should be interpreted alongside rank-based testing.

### 2.5 Statistical analysis

Continuous variables are presented as median with interquartile range (IQR) and as mean with standard deviation (SD) where relevant. Paired within-patient comparisons between baseline and month-12 values were performed using the Wilcoxon signed-rank test. Between-group comparisons of continuous variables were performed using the Mann-Whitney *U* test; categorical variables were compared using Fisher’s exact test. Effect size for paired comparisons was estimated using Cohen’s *d* for paired samples. Ninety-five percent confidence intervals for mean differences were calculated using the paired *t*-distribution.

Correlations between continuous variables were assessed using both Spearman rank correlation (ρ) and Pearson correlation (*r*). Spearman rank correlation was the pre-specified primary method given the marked positive skewness and non-normal distribution of SII and plasma HIV RNA. Pearson correlation was examined as a sensitivity analysis and is reported alongside Spearman estimates to facilitate comparison. Where Pearson and Spearman estimates diverged substantially — defined as directional agreement but a >2-fold difference in the magnitude of the coefficient, or discordant significance — the source of discrepancy was investigated by identifying high-leverage data points through visual inspection of scatter plots and by repeating the analysis after sequential exclusion of the one or two most extreme values. Log_1__0_ transformation of plasma HIV RNA was applied as a further sensitivity analysis for correlation analyses involving viral load.

Where paired correlation coefficients from the same cohort were compared across time points, the Steiger *z*-test for dependent correlations (Meng, Rosenthal & Rubin, 1992) was applied to formally assess whether the coefficients differed significantly.

Age-stratified analyses were performed using the Kruskal–Wallis test with *post-hoc* pairwise Mann–Whitney U testing. Because the overall Kruskal–Wallis test was not statistically significant and no adjustment for multiple pairwise comparisons was applied, *post-hoc* p values were considered exploratory and hypothesis-generating rather than confirmatory. Nutritional status was evaluated using the Gomez classification, in which a weight-for-age percentage ≥90% was considered normal and values below this threshold were classified as Grade 1 (76–89%), Grade 2 (61–75%), or Grade 3 (<61%) malnutrition. No adjustment for multiple comparisons was applied to the secondary exploratory analyses presented in Sections 3.4–3.8; corresponding *p*-values should therefore be considered nominal.

All tests were two-sided, and a *p*-value of <0.05 was considered statistically significant. Given the exploratory nature of the study and the limited sample size (*n* = 30), all *p*-values should be interpreted descriptively, and statistically significant findings should be regarded as hypothesis-generating rather than confirmatory, warranting further investigation in adequately powered prospective studies. All statistical analyses were performed using Python with the SciPy and NumPy libraries.

## 3 Results

### 3.1 Patient characteristics

The study cohort comprised 30 pediatric patients with confirmed HIV infection (20 males [66.7%], 10 females [33.3%]), with a median age of 12.5 years (IQR 4.4–15.8; range 0.02–17.0 years). HIV was acquired through vertical transmission in 11 patients (36.7%) and via blood, dental, or surgical exposure in 7 (23.3%); transmission route in the remaining 12 patients (40.0%) was consistent with the adolescent age profile of this cohort, given that 16 of 30 patients (53.3%) were aged 12 years or older at the time of evaluation. By immunological staging, 14 patients (46.7%) were classified as immunological stage 1, 9 (30.0%) as stage 2, and 7 (23.3%) as stage 3 at baseline.

With regard to antiretroviral regimens, 20 patients (66.7%) received an integrase strand transfer inhibitor (INSTI)-based regimen and 10 (33.3%) a protease inhibitor (PI)-based regimen. Among INSTI-based regimens, Genvoya^®^ (EVG/COBI/TAF/FTC) was the most commonly prescribed agent (*n* = 11, 36.7%), followed by Biktarvy^®^ (BIC/TAF/FTC; *n* = 5, 16.7%), DTG-based regimens (*n* = 3, 10.0%), and raltegravir-based (*n* = 1, 3.3%). The PI-based group comprised lopinavir/ritonavir-containing regimens (*n* = 10, 33.3%). Two patients (6.7%) received valganciclovir during the follow-up period.

Nutritional status was assessed using both the Gomez classification and body mass index (BMI). Median BMI was 17.2 kg/m² (IQR 15.3–22.8; range 9.4–26.8). By Gomez classification, 21 patients (70.0%) had a normal weight-for-age (≥90%), and 9 (30.0%) were classified as malnourished, of whom 6 had Grade 1 (76–89%), 1 Grade 2 (61–75%), and 2 Grade 3 (<61%) malnutrition. Patients with an abnormal Gomez score received supplemental enteral nutritional support as part of standard clinical care.

### 3.2 Overall SII and CD4 trajectory from baseline to month 12

At baseline, median HIV RNA was 39,796 copies/mL (IQR 136–619,028), with 13 patients (43.3%) presenting with viral loads below 10,000 copies/mL, 3 (10.0%) between 10,000 and 100,000 copies/mL, and 14 (46.7%) exceeding 100,000 copies/mL. Median CD4 percentage was 25.0% (IQR 16.0–32.8), median absolute CD4 count was 655 cells/µL (IQR 507–1,175), and median SII was 342,637 (IQR 164,882–406,417).

Of the 30 patients with complete paired data, median SII declined numerically from 342,637 at baseline to 250,527 at month 12 (difference of medians: −92,110; mean paired difference: 103,669 [95% CI −118,970 to 326,308]; Cohen’s d = 0.174). Note that the difference of medians and the mean paired difference are distinct quantities: the former subtracts the two group medians directly, whereas the latter represents the mean of individual within-patient changes. Both are reported for completeness; the Wilcoxon signed-rank test (which uses individual paired differences) is the pre-specified primary inferential method. This change did not reach statistical significance on either paired t-test (t = 0.952, *p* = 0.349) or Wilcoxon signed-rank test (W = 193.0, *p* = 0.428). The absence of a significant overall change in SII reflected bidirectional individual trajectories: 17 of 30 patients (56.7%) demonstrated a decline, whereas 13 (43.3%) showed an increase or no change. In contrast, CD4 percentage improved significantly over the same period (median 25.0% to 35.0%, Wilcoxon *p* < 0.001), as did absolute CD4 count and HIV RNA (**Table 1**).

**Table 1:** Changes in SII, CD4 percentage, CD4 count, and HIV RNA from baseline to month 12 (n = 30). .

Variable	Baseline median (IQR)	Month 12 median (IQR)	Median change (month 12 − baseline)	*p*
SII	342,637 (164,882–406,417)	250,527 (141,179–418,153)	−92,110	0.428
CD4 (%)	25.0 (16.0–32.8)	35.0 (29.0–37.0)	+10.0	< 0.001 *
CD4 count (cells/µL)	655 (507–1,175)	1,021 (732–1,371)	+366	0.015 *
HIV RNA (copies/mL)	39,796 (136–619,028)	10 (10–163)	—	< 0.001 *
DNT (%)	6.2 (4.9–7.0)	5.8 (5.0–7.5)	−0.4	0.734

Wilcoxon signed-rank test. *p < 0.05 is shown in bold. IQR, interquartile range. The ‘Median change’ column reports the arithmetic difference of medians (Month-12 median minus Baseline median). The mean reduction of 103,669 cited in the text refers to the mean of individual paired within-patient differences, which is a distinct quantity; both are reported for completeness. SII values reflect calculations performed using absolute blood cell counts per µL.

Individual SII components were examined to characterize the observed trajectory. Neutrophil, lymphocyte, and platelet counts each showed non-significant changes over 12 months (*p* = 0.178, 0.339, and 0.428, respectively; **Table 2**), suggesting that the overall absence of SII change reflects modest, concurrent shifts across all three lineages rather than a dominant single-component effect.

**Table 2:** Longitudinal trajectories of individual SII components from baseline to month 12 (n = 30).

Variable	Baseline	Month 12	*p*-value
Neutrophil count (cells/µL)	2,850 (2,400–4,240)	2,465 (1,960–3,445)	0.178
Lymphocyte count (cells/µL)	2,550 (1,978–3,370)	2,710 (2,105–3,750)	0.339
Platelet count (cells/µL)	260,000 (203,000–313,000)	280,000 (241,000–346,000)	0.428

Values are median (interquartile range). p-values from Wilcoxon signed-rank test.

DNT% did not change significantly over 12 months (baseline 6.2% [IQR 4.9–7.0] vs. month-12 5.8% [IQR 5.0–7.5]; *p* = 0.734), and no significant correlation was observed between ΔSII and ΔDNT% (Spearman ρ = +0.124, *p* = 0.512).

### 3.3 Dissociation of SII from CD4 percentage after ART initiation

At baseline, SII correlated inversely with CD4 percentage on Pearson analysis (*r* = −0.450, *p* = 0.013), though this association did not reach significance by the pre-specified Spearman method (ρ = −0.296, *p* = 0.113). At month 12, both estimates were negligible (Pearson *r* = −0.004; Spearman ρ = +0.015), and a Steiger z-test confirmed that the Pearson coefficients at the two time points differed significantly (*z* = −4.889, *p* < 0.001) only (**Table 3**). These findings indicate substantial attenuation of the SII–CD4% relationship following ART initiation, consistent with SII and CD4 percentage reflecting increasingly distinct biological processes over time.

**Table 3:** Pearson and Spearman correlation between SII and CD4 percentage at baseline and month 12 (n = 30).

Timepoint	Pearson *r*	*p* (Pearson)	Spearman ρ	*p* (Spearman)	n
Baseline	−0.450	0.013 *	−0.296	0.113	30
Month 12	−0.004	0.985	+0.015	0.937	30

*p < 0.05 is shown in bold.

### 3.4 Relationship between HIV RNA decline and SII change

The analyses presented in Sections 3.4–3.8 are secondary and exploratory. No correction for multiplicity has been applied; all findings should be regarded as hypothesis-generating rather than confirmatory. No robust association was found between SII and HIV RNA. The Pearson correlation between paired SII and HIV RNA changes was nominally significant (*r* = +0.534, *p* = 0.002), but this was driven by two patients with extremely high baseline viral loads and disappeared on rank-based testing, log-transformation, and outlier exclusion. Neither virological suppression nor the direction of viral load change predicted SII trajectory. The same pattern was seen in cross-sectional analyses at both time points: Pearson and Spearman estimates diverged, and no association survived outlier exclusion. For the baseline cross-sectional analysis, the Pearson coefficient was *r* = +0.471 (*p* = 0.009) but fell to *r* = −0.187 (*p* = 0.340) after exclusion of the two high-leverage patients, with a non-significant Spearman estimate throughout (ρ = −0.084, *p* = 0.657) (**Table 4**).

**Table 4:** Correlation analyses between HIV RNA and SII at baseline and over 12 months (n = 30).

Analysis	Pearson *r*	*p*	Spearman ρ	*p*	n
ΔSII ~ ΔHIV RNA	+0.534	0.002 *	+0.004	0.982	30
ΔSII ~ Δlog_1__0_(HIV RNA)	+0.196	0.299	−0.056	0.769	30
Baseline SII ~ Baseline HIV RNA	+0.471	0.009 *	−0.084	0.657	30
Baseline SII ~ Baseline HIV RNA (outliers excluded)	−0.187	0.340	−0.096	0.627	28
Month-12 SII ~ Month-12 HIV RNA	−0.177	0.351	−0.177	0.351	30
ΔSII ~ ΔHIV RNA (outliers excluded)	+0.096	0.629	−0.045	0.818	28

*p < 0.05. Spearman rank-based testing used as the primary inferential method given the non-normal distribution of HIV RNA values. Outlier-excluded analysis removed two patients with extreme baseline viremia (>9 × 10^6^ copies/mL).

Bold values indicate statistically significant results (p < 0.05).

### 3.5 Characterization of SII non-improvers

#### 3.5.1 Continuous variables

Baseline SII (387,818 vs. 314,294; *p* = 0.004) and neutrophil count (4,000 vs. 2,400 cells/µL; *p* = 0.006) were nominally higher in SII-improvers, both findings should be interpreted as mathematical consequences of the outcome definition rather than independent biological predictors: they do not constitute evidence of a predictive association and are reported here for descriptive completeness only (**Table 5**).

**Table 5:** Comparison of baseline continuous variables between SII-improved and SII-not-improved groups.

Variable	SII improved (*n* = 17) median	SII not improved (*n* = 13) median	Difference	*p*
Age (years)	14.0	15.0	−1.0	0.567
Baseline SII	387,818	314,294	+73,524	0.004 *
Baseline WBC (cells/µL)	6,210	5,400	+810	0.052
Baseline Neutrophil (cells/µL)	4,000	2,400	+1,600	0.006 *
Baseline Lymphocyte (cells/µL)	2,600	2,500	+100	0.917
Baseline Platelet (cells/µL)	262,300	253,000	+9,300	0.161
Baseline CD4 (%)	24.0	25.0	−1.0	0.503
Baseline CD4 (cells/µL)	572	670	−98	0.568
Baseline CD4/CD8 ratio	0.50	0.60	−0.10	0.828
Baseline HIV RNA	369,900	38,459	+331,441	0.503
Gomez % (nutritional)	95.2	97.0	−1.8	0.770
BMI (kg/m²)	16.6	19.0	−2.4	0.851

*p < 0.05 is shown in bold; Mann-Whitney U test. Values are medians.

#### 3.5.2 Categorical variables

Among categorical variables female sex was more frequent in the SII-not-improved group (7/13, 53.8% vs. 3/17, 17.6%), although the difference did not reach statistical significance (Fisher’s exact *p* = 0.056) (**Table 6**). This finding should be interpreted with caution given the multiplicity of comparisons performed. Genvoya^®^ (EVG/COBI/TAF/FTC) was numerically more frequent among non-improvers (7/13, 53.8% vs. 4/17, 23.5%), although this difference did not reach statistical significance (*p* = 0.132). Virologic suppression at month 12 (HIV RNA ≤20 copies/mL) did not differ between groups (64.7% vs. 53.8%; *p* = 0.711). Transmission route, CMV DNAaemia, EBV DNAaemia, malnutrition grade, and valganciclovir use were also not significantly associated with SII trajectory.

**Table 6:** Comparison of categorical variables between SII-improved and SII-not-improved groups.

Variable	SII improved *n* (%)	SII not improved *n* (%)	*p* (Fisher’s exact)
Female sex	3/17 (17.6%)	7/13 (53.8%)	0.056
Vertical transmission	7/17 (41.2%)	4/13 (30.8%)	0.708
Sexual transmission	7/17 (41.2%)	5/13 (38.5%)	1.000
Genvoya^®^ (EVG/COBI/TAF/FTC)	4/17 (23.5%)	7/13 (53.8%)	0.132
CMV-positive at baseline **	1/14 (7.1%)	3/11 (27.3%)	0.288
EBV-positive at baseline **	4/15 (26.7%)	4/12 (33.3%)	1.000
Any herpesvirus co-infection **	4/15 (26.7%)	6/12 (50.0%)	0.257
Virologic suppression (≤20 c/mL)	11/17 (64.7%)	7/13 (53.8%)	0.711
Malnutrition (Gomez class 1–3)	5/17 (29.4%)	4/13 (30.8%)	1.000

*p < 0.05; Fisher’s exact test. EVG = elvitegravir; COBI, cobicistat; TAF, tenofovir alafenamide; FTC, emtricitabine.

**For CMV, EBV, and herpesvirus co-infection rows, denominators are restricted to evaluable patients (CMV: n=25 evaluable [5 with missing DNAaemia excluded]; EBV: n=27 [3 excluded]; any herpesvirus: n=27 [3 excluded]).

### 3.6 Age-stratified analysis

Age-stratified analyses did not identify a significant overall difference in SII trajectory across the five age groups (Kruskal–Wallis H = 6.804, *p* = 0.147). The 1–2-year age group had the highest proportion of SII non-improvement, with 3 of 4 patients (75.0%) showing no improvement only (**Table 7**). In an exploratory unadjusted *post-hoc* comparison, the 1–2-year group differed from the 6–11-year group (Mann–Whitney *p* = 0.038). Given the non-significant overall test, the absence of correction for multiple comparisons, and the small number of patients in the 1–2-year subgroup, this finding should be interpreted as exploratory rather than as evidence of a definitive age-group difference.

**Table 7:** SII trajectory stratified by age group (n = 30). Kruskal-Wallis overall p = 0.147.

Age group	*n*	SII improved	SII not improved	% Not improved	Median ΔSII
< 1 year	2	2 (100%)	0 (0%)	0%	−159,956
1–2 years	4	1 (25%)	3 (75%)	75%	+80,850
3–5 years	2	1 (50%)	1 (50%)	50%	+13,888
6–11 years	6	5 (83%)	1 (17%)	17%	−323,903
12–17 years	16	8 (50%)	8 (50%)	50%	−49,162

Overall Kruskal–Wallis *p* = 0.147. Post-hoc Mann–Whitney comparisons: 1–2 years vs. 6–11 years, *p* = 0.038; 6–11 years vs. 12–17 years, *p* = 0.049. Post-hoc *p* values were not adjusted for multiple comparisons and should be interpreted as exploratory.

Fisher’s exact test revealed that neutrophil counts below 1,500 cells/µL were significantly more prevalent in patients aged less than 6 years compared to older patients (4/8 [50.0%] vs. 0/22 [0.0%]; *p* = 0.003), suggesting an age-dependent pattern of neutrophil abundance that may partly underlie this finding. Since neutrophil count is the primary numerator of the SII formula, children with lower baseline neutrophil counts necessarily start with a lower absolute SII, limiting their mathematical room for decline; the observed SII pattern in younger children may therefore reflect, at least in part, developmental differences in neutrophil abundance and the mathematical properties of the SII formula; these contributions cannot be disentangled in the present dataset. A second pairwise comparison reached nominal significance: the 6–11 year group showed a better SII response than the 12–17 year group (Mann-Whitney *p* = 0.049). On closer examination, however, this finding is driven by a single patient with an extreme baseline SII of 2,969,372 and a correspondingly large absolute decline (ΔSII −2,775,829), illustrating the same regression-to-the-mean effect described above. The remaining five patients in the 6–11 year group had a median ΔSII of −220,802, which is not meaningfully different from the 12–17 year group median of −49,162. This comparison is therefore not considered to reflect a true biological advantage at 6–11 years and should not be interpreted as evidence of a developmental “sweet spot” without replication in a larger cohort.

### 3.7 Herpesvirus co-infections and SII trajectory

Herpesvirus co-infection status was not significantly associated with SII trajectory. Neither baseline CMV DNAaemia (75.0% vs. 38.1% SII non-improvement in CMV-detectable vs. CMV-undetectable patients; Fisher’s exact *p* = 0.288) nor EBV DNAaemia (50.0% vs. 42.1%; *p* = 1.000) was significantly associated with SII trajectory only (**Table 8**). CMV and EBV status were determined by quantitative real-time PCR (COBAS 6800 platform, Roche Diagnostics); values below the lower limit of quantification of the respective assay (<34.5 or <79.4 copies/mL depending on assay generation) were classified as undetectable DNAaemia. Viral load positivity was used as a binary classification tool (detectable vs. undetectable DNAaemia) rather than as a marker of end-organ disease. No patient in this cohort was clinically diagnosed with CMV or EBV end-organ involvement during the observation period. CMV-detectable patients exhibited a markedly worse median ΔSII (+76,780 vs. −142,302; Mann-Whitney *p* = 0.262), and patients with any herpesvirus co-infection had a numerically worse SII response than co-infection-negative patients (6/10, 60.0% vs. 6/17, 35.3%; *p* = 0.257) (**Table 8**). CMV and EBV DNAaemia status could not be determined for 5 and 3 patients, respectively, owing to missing baseline data. Over the 12-month period, viral clearance (undetectable DNAaemia at month 12) occurred in 3 of 4 evaluable CMV-positive and 4 of 8 EBV-positive patients. Only two patients received valganciclovir; both demonstrated SII improvement at month 12 (ΔSII −215,668 and −220,802, respectively). For Patient 1 (valganciclovir 12 months), all hematological parameters were stable or increased at month 12, providing no evidence of myelosuppression. For Patient 20 (valganciclovir 16 months, with the 12-month draw during active exposure), neutrophil count declined (4,300 → 2,020/µL); however, concurrent declines in lymphocyte, platelet, and total WBC counts suggest overall CBC normalization from a markedly elevated baseline rather than selective drug-induced myelosuppression. A drug contribution cannot be formally excluded; both findings are reported descriptively without causal interpretation.

**Table 8:** Herpesvirus co-infection status and SII trajectory (n = 30).

Variable	*n*	SII improved	SII not improved	Median ΔSII	*P (Fisher’s exact)*
CMV-positive (baseline)	4	1 (25.0%)	3 (75.0%)	+76,780	0.288
CMV-negative (baseline)	21	13 (61.9%)	8 (38.1%)	−142,302	—
EBV-positive (baseline)	8	4 (50.0%)	4 (50.0%)	+16,533	1.000
EBV-negative (baseline)	19	11 (57.9%)	8 (42.1%)	−142,302	—
Any herpesvirus co-infection	10	4 (40.0%)	6 (60.0%)	+41,952	0.257
No herpesvirus co-infection	17	11 (64.7%)	6 (35.3%)	−176,247	—
Valganciclovir use*	2	2 (100%)	0 (0%)	−218,235	N/A

Mann-Whitney U test for ΔSII comparisons. Fisher’s exact test for categorical comparisons. N/A = group too small for statistical testing. CMV and EBV analyses restricted to patients with evaluable DNAaemia data: CMV (n = 25 evaluable; 5 patients excluded), EBV (n = 27 evaluable; 3 patients excluded). Patients with missing data are not counted as negative.

*Patient 1: all CBC parameters stable or increased (neutrophil 2,400 → 2,700/µL); myelosuppression unlikely. Patient 20: pan-lineage CBC decline (neutrophil 4,300 → 2,020/µL; lymphocyte 4,700 → 3,570/µL; platelet 451,000 → 339,000/µL) consistent with baseline normalization; drug contribution cannot be excluded.

### 3.8 Nutritional status and SII trajectory

Nutritional status, as assessed by Gomez percentage, was not associated with SII trajectory. Median Gomez percentage did not differ between groups (95.2% vs. 97.0%; Mann-Whitney *p* = 0.770), and no linear association was observed between Gomez percentage and the magnitude of SII change (Pearson *r* = 0.230, *p* = 0.222; Spearman ρ = 0.189, *p* = 0.317). The proportion of SII non-improvement was nearly identical in patients with normal vs. abnormal Gomez scores (42.9% vs. 44.4%; Fisher’s exact *p* = 1.000), and stratification by malnutrition grade (Gomez class 1–3 vs. normal) likewise yielded no significant difference (*p* = 1.000). BMI did not differ between groups (median 16.6 vs. 19.0 kg/m²; *p* = 0.851).

## 4 Discussion

The principal finding of this study is that systemic immune-inflammation index (SII) failed to improve in nearly half (43.3%) of pediatric patients with HIV after 12 months of antiretroviral therapy, despite significant concurrent improvement in CD4 percentage and virologic suppression. This observation underscores a fundamental dissociation between conventional markers of treatment success — CD4 reconstitution and HIV RNA suppression — and the inflammatory milieu as quantified by SII. Our data are consistent with the broader literature documenting residual immune activation in virologically suppressed people with HIV (Lederman et al., 2013), and extend those observations to a pediatric cohort in which the determinants of SII response appear to be multifactorial and largely independent of viral load dynamics.

The robust suppression of HIV RNA observed at month 12 (median decline from 39,796 to 10 copies/mL, *p* < 0.001) was not accompanied by a corresponding significant reduction in SII (*p* = 0.428). Furthermore, virologic suppression status at month 12 did not predict SII direction, and among patients with declining HIV RNA, SII increased paradoxically in 44.0%. Although Pearson analysis suggested a positive correlation between ΔSII and ΔHIV RNA (*r* = +0.534, *p* = 0.002), this association was not confirmed by rank-based testing (Spearman ρ = +0.004) and by log_1__0_ transformation of HIV RNA, confirming that it reflected the leverage of two patients with extreme baseline viremia rather than a true biological relationship. Collectively, these findings indicate that suppression of circulating viremia may be necessary but is not sufficient for normalization of the SII-reflected inflammatory milieu. The discrepant trajectories likely reflect residual immune activation, viral reservoir persistence, microbial translocation, ART-specific hematologic effects, or co-infections that are not fully captured by plasma HIV RNA alone (Brenchley et al., 2006; Chun and Fauci, 2012; Deeks et al., 2013b; Lederman et al., 2013; Marchetti et al., 2013; Masiá et al., 2014; Massanella et al., 2016; Nkambule et al., 2020; Madzime et al., 2021; Serrano-Villar et al., 2022). It should also be noted that baseline blood draws were obtained at the time of HIV diagnosis, a clinical context in which intercurrent illness is common in this population; the elevated baseline SII values observed in this cohort therefore reflect not only untreated HIV-related inflammation but also the broader clinical burden present at diagnosis.

This interpretation is supported by Wang et al., who reported that SII levels in virologically suppressed people with HIV were paradoxically lower than those without HIV, attributing this to the altered hematologic context imposed by HIV disease and ART rather than systemic inflammatory burden per se (Wang et al., 2025). Our data reinforce and extend this conclusion by demonstrating that even within a treated HIV cohort, SII trajectories diverge substantially and cannot be predicted by virological endpoints alone.

SII-improvers had nominally higher baseline SII (387,818 vs. 314,294, *p* = 0.004) and neutrophil counts (4,000 vs. 2,400 cells/µL, *p* = 0.006). Both findings are most parsimoniously attributed to regression to the mean: higher baseline values carry greater mathematical room for decline, and the neutrophil count is itself the primary numerator of the SII formula. A biological interpretation — that neutrophil-driven baseline inflammation may be more amenable to ART-mediated rebalancing — remains plausible given prior evidence that neutrophil dynamics in HIV are influenced by ART class independently of virological control (Nkambule et al., 2020; Madzime et al., 2021), but cannot be formally distinguished from mathematical coupling in a cohort of this size. These observations are therefore retained as hypothesis-generating rather than confirmatory. Accordingly, neither baseline SII nor baseline neutrophil count should be regarded as a clinical predictor of SII response in this cohort; both associations are artefacts of the outcome definition and the mathematical structure of the SII formula.

The substantial attenuation of the SII–CD4% correlation between baseline (Pearson *r* = −0.450, *p* = 0.013; Spearman ρ = −0.296, *p* = 0.113) and month 12 (Pearson *r* = −0.004, *p* = 0.985; Steiger *z* = −4.889, *p* < 0.001 for the difference between coefficients) is among the most biologically informative findings of this study. At baseline, lower CD4 percentage correlated with higher SII, consistent with the established link between immunosuppression and systemic inflammation in untreated HIV. However, after ART initiation, CD4 reconstitution proceeded along a trajectory that became progressively dissociated from SII. CD4 reconstitution reflects the restoration of lymphopoiesis and the withdrawal of direct viral cytopathic effects (Lewis et al., 2017) — a process that responds relatively promptly to viremia suppression. SII, by contrast, integrates neutrophil and platelet dynamics alongside lymphocyte counts; these components are less directly governed by viral replication and may instead be modulated by microbial translocation, viral reservoirs, co-infections, or ART-specific hematologic effects. Our findings therefore reinforce the interpretation that in treated pediatric HIV, SII and CD4 percentage reflect distinct biological states, and that SII may serve as a complementary rather than redundant inflammatory biomarker.

The stability of DNT% despite virological suppression is consistent with the broader pattern of incomplete immune normalization observed in this cohort, and its independence from SII trajectory suggests that these two biomarkers capture distinct aspects of residual immune dysregulation.

Female sex was more frequent among patients with SII non-improvement (53.8% vs. 17.6%), although this difference did not reach statistical significance (*p* = 0.056). Although the small sample size warrants caution, this finding is biologically plausible. Sex-based differences in innate and adaptive immunity are well established, with females generally exhibiting stronger type I interferon signaling, higher monocyte activation, and more robust antiviral responses (Meier et al., 2009; Jiang et al., 2014; Klein and Flanagan, 2016). In the context of HIV, these immunological sex differences may sustain a higher inflammatory tone even under viral suppression. Additionally, sex-specific differences in neutrophil and platelet function — both key SII components — have been described and may contribute to the divergent SII trajectories observed here. Prospective studies in larger, sex-stratified pediatric cohorts are needed to evaluate this association.

Integrase strand transfer inhibitors (INSTIs), including Genvoya^®^ (EVG/COBI/TAF/FTC), have become a preferred component of first-line pediatric antiretroviral regimens given their favorable efficacy and tolerability (Messiaen et al., 2013). The numerical over-representation of Genvoya^®^ among SII non-improvers (**Table 6**) did not reach statistical significance in this cohort (*p* = 0.132), but is consistent with prior evidence that different ART regimens exert distinct effects on circulating inflammatory mediators and hematologic parameters independent of virologic response (Massanella et al., 2014; Drozd et al., 2016; Serrano-Villar et al., 2022) and that neutrophil and platelet dynamics in HIV may be influenced by ART class rather than viral load alone (Nkambule et al., 2020; Madzime et al., 2021). Whether cobicistat-mediated CYP3A inhibition or EVG/TAF-specific intracellular effects modulate the neutrophil, platelet, or lymphocyte kinetics that constitute the SII formula remains an open question not yet addressed in the literature and warrants dedicated prospective investigation.

Age-stratified analyses did not provide evidence of an overall age-related difference in SII trajectory (Kruskal–Wallis *p* = 0.147). Nevertheless, the 1–2-year age group had the highest proportion of SII non-improvement, with 3 of 4 patients (75.0%) showing no improvement. An unadjusted *post-hoc* comparison between the 1–2-year and 6–11-year groups yielded a nominal *p* value of 0.038. This observation should be interpreted cautiously, however, because the overall age-group comparison was not statistically significant, no correction for multiple pairwise comparisons was applied, and the 1–2-year subgroup included only four patients. Moreover, all three non-improving patients in this age group had vertically acquired HIV and were receiving PI-based regimens, further limiting attribution of the observed pattern to age alone. Therefore, these findings should be regarded as exploratory and hypothesis-generating rather than as evidence that children aged 1–2 years constitute a distinct vulnerable subgroup. Larger prospectively stratified pediatric cohorts are required to determine whether age or developmental stage independently influences SII dynamics after ART. The nominally significant 6–11 vs. 12–17 year comparison (*p* = 0.049) was examined in detail and is not considered independently interpretable. The 6–11 year group contained one patient with an extreme baseline SII of 2,969,372 whose large absolute decline disproportionately drove the group median and the Mann-Whitney statistic; the remaining patients in this stratum showed responses comparable to those of the adolescent group. This is a further illustration of the regression-to-the-mean phenomenon that limits interpretation of all between-group comparisons of SII change in this cohort. However, the directionality of this comparison is biologically plausible: children aged 6–11 years in this cohort presented with higher baseline viral loads and SII values, potentially providing greater absolute room for ART-mediated SII reduction. The mechanisms underlying the comparatively attenuated response in the adolescent group remain unclear and warrant prospective evaluation in larger cohorts. Although no statistically significant association was identified between herpesvirus co-infection status and SII trajectory, CMV-positive patients (defined as those with detectable CMV DNAaemia by quantitative PCR on the COBAS 6800 platform) showed a numerically higher rate of SII non-improvement (75.0%) and a markedly worse median ΔSII (+76,780 vs. −142,302; *p* = 0.262), and patients with any herpesvirus co-infection showed a similar trend (60.0% vs. 35.3%, *p* = 0.257). Viral load positivity was used here as a binary classification tool (detectable vs. undetectable DNAaemia) rather than as a marker of end-organ disease; no patient in this cohort was clinically diagnosed with CMV or EBV end-organ involvement during the observation period. Continuous quantitative viral load values were available but were not used as continuous predictors given the small number of detectable cases (*n* = 4 for CMV, *n* = 8 for EBV) and the resulting lack of statistical power for regression-based analyses. These differences, likely underpowered given the small number of CMV-positive patients (*n* = 4), are directionally consistent with the known capacity of CMV to sustain immune activation through persistent T-cell and monocyte stimulation (Gianella and Letendre, 2016; Tavenier et al., 2019; Schnittman and Hunt, 2021; Rinaldo, 2022). Interpretation is further limited by the small number of valganciclovir recipients (*n* = 2, both of whom improved) and by missing baseline DNAaemia data in a substantial proportion of patients (5 of 30 for CMV, 3 of 30 for EBV). These findings suggest a possible but unconfirmed contribution of herpesvirus activity to sustained SII elevation, warranting evaluation in adequately powered studies with complete co-infection screening and systematic documentation of end-organ disease.

In contrast to the above factors, nutritional status — assessed by Gomez percentage and malnutrition grade — showed no significant association with SII trajectory at any level of analysis (all *p* > 0.05). This null finding warrants cautious interpretation: patients with an abnormal Gomez score received supplemental enteral nutritional support as part of standard care, an intervention capable of correcting the hematologic parameters that constitute the SII formula. The absence of an observed association may therefore reflect successful clinical correction of nutritional deficits during follow-up rather than a true absence of biological relationship. Disentangling these possibilities would require stratifying malnourished patients by receipt versus non-receipt of nutritional intervention, or using baseline micronutrient markers unaffected by subsequent treatment — neither of which was feasible within the present dataset.

Several limitations of this study warrant acknowledgement. The retrospective single-center design limits generalizability, and the sample size (*n* = 30) restricts statistical power for subgroup analyses and multivariable adjustment. In particular, the 1–2-year age subgroup comprised only four patients, substantially limiting the reliability and generalizability of the age-stratified comparisons. The observational design precludes causal inference, and all secondary findings should be regarded as hypothesis-generating pending replication in larger prospective studies. No formal correction for multiple comparisons was applied to the secondary analyses in Sections 3.4–3.8; nominal *p*-values should be interpreted with caution, as some findings reaching *p*< 0.05 may represent false positives.

Baseline blood draws were obtained at the time of HIV diagnosis, a clinical context in which intercurrent illness is common and inherently reflected in SII values. Twelve-month assessments were conducted during scheduled routine follow-up visits; clinical records do not document active intercurrent or opportunistic infection at the time of these assessments, Although IRIS has been described in infants initiating ART (Smith et al., 2009), no patient in the present cohort was diagnosed with IRIS during the observation period. Although IRIS has been described in infants initiating ART (Smith et al., 2009), no patient in the present cohort was diagnosed with IRIS during the observation period. The majority of patients were older than 2 years at diagnosis and had completed the intensive early childhood vaccination schedule; however, the timing of 12-month blood draws relative to any intercurrent booster vaccinations was not systematically recorded.

No patient underwent a planned treatment interruption; a small number experienced brief gaps in drug supply not exceeding one month in duration, which were not considered formal treatment discontinuations and are unlikely to have materially affected the 12-month outcomes.

The enteral nutritional support received by patients with abnormal Gomez scores represents a potential confounder for the Gomez–SII analysis, as nutritional intervention may itself influence hematologic parameters. The cross-sectional nature of the 12-month assessment precludes characterization of the kinetics of SII change over time.

Co-infection analyses were limited by missing DNAaemia data for CMV (*n* = 5) and EBV (*n* = 3); patients with missing data were excluded from co-infection analyses rather than classified as negative. The binary classification of herpesvirus status as detectable versus undetectable DNAaemia did not account for viral load magnitude, and the small number of CMV-detectable patients (*n* = 4) precluded quantitative viral load analyses.

Finally, the observed numerical signals for female sex and Genvoya^®^ use in relation to SII non-improvement are hypothesis-generating and require confirmation in larger prospective cohorts with pre-specified stratification by these variables.

## 5 Conclusion

In this cohort of ART-treated pediatric patients with HIV, SII failed to improve in 43.3% of cases despite significant cohort-level virological suppression and CD4 reconstitution. SII trajectories were dissociated from both HIV RNA dynamics and CD4 percentage after 12 months of ART, underscoring that SII reflects a distinct immunological dimension not captured by conventional treatment endpoints. Higher baseline SII and neutrophil counts were associated with greater SII reductions, although these findings likely reflect regression to the mean and mathematical coupling. A numerical difference according to sex was observed, but it did not reach statistical significance, whereas age-, ART regimen-, and herpesvirus-related findings remained exploratory and require confirmation in larger prospective cohorts. Overall, these findings suggest that SII may capture a residual inflammatory dimension of treated pediatric HIV that is not fully reflected by conventional virological and immunological endpoints.

## Data Availability

The raw data supporting the conclusions of this article will be made available by the authors, without undue reservation.
